# Transcranial Direct Current Stimulation (tDCS) in Diabetes: A Focused and Mechanistic Review of Symptom and Function Outcomes

**DOI:** 10.3390/jcm14227945

**Published:** 2025-11-09

**Authors:** James Chmiel, Donata Kurpas

**Affiliations:** 1 Institute of Neurofeedback and TDCS Poland, 70-393 Szczecin, Poland; 2 Faculty of Health Sciences, Department of Family and Pediatric Nursing, Wrocław Medical University, 51-618 Wrocław, Poland

**Keywords:** tDCS, transcranial direct current stimulation, non-invasive brain stimulation, neurostimulation, neuromodulation, diabetes, diabetic, diabetes mellitus

## Abstract

Transcranial direct current stimulation (tDCS) is being explored as an adjunct for diabetes-related symptoms grounded in diabetes-associated alterations in brain networks. We reviewed clinical trials of tDCS conducted in people with diabetes and summarized mechanistic findings relevant to metabolic control. Two reviewers searched PubMed/MEDLINE, Cochrane Library, Google Scholar, Scopus, and ResearchGate for studies published from 1 January 2008 to 31 August 2025. Forty-one records were identified; after screening and full-text assessment, 11 studies met the inclusion criteria. Across predominantly middle-aged adults with long-standing type 2 diabetes, protocols were low-intensity and well-tolerated. The most consistent clinical benefit was analgesia with primary motor cortex stimulation, with randomized comparisons favoring active tDCS over sham. Dorsolateral prefrontal stimulation paired with working-memory training improved cognition and reduced anxiety, while combined motor–prefrontal courses yielded gains in sleep quality and health-related quality of life; a small, randomized study in proliferative diabetic retinopathy reported short-term visual improvements after occipital stimulation. Safety was favorable, and no serious adverse events were reported. Objective metabolic endpoints in diabetic cohorts were scarce; early evidence for insulin-independent improvements in glucose handling and neurometabolic shifts derives mainly from non-diabetic or mixed samples and remains hypothesis-generating. Overall, tDCS appears to be a promising, well-tolerated adjunct for diabetes-related complications. Larger, rigorously sham-controlled trials that align targets with clinical phenotypes and include standardized metabolic outcomes are needed.

## 1. Introduction

Diabetes mellitus is a heterogeneous group of chronic metabolic disorders defined by persistent hyperglycemia due to defects in insulin secretion, insulin action, or both [1,2]. The principal clinical categories are type 1 diabetes (autoimmune β-cell destruction leading to absolute insulin deficiency), type 2 diabetes (a combination of insulin resistance and progressive β-cell dysfunction), gestational diabetes mellitus (GDM) diagnosed in the second or third trimester, and “other specific types” (e.g., monogenic diabetes, pancreatogenic diabetes, cystic-fibrosis–related diabetes, and drug- or endocrine-induced hyperglycemia) [3]. Diagnostic thresholds are standardized internationally—diabetes may be diagnosed by fasting plasma glucose ≥ 126 mg/dL (7.0 mmol/L), 2 h plasma glucose ≥ 200 mg/dL (11.1 mmol/L) during a 75 g OGTT, or HbA1c ≥ 6.5%—with confirmatory testing in the absence of unequivocal hyperglycemia [2,3,4].

The global burden of diabetes has risen dramatically over the last three decades. A 2024 analysis of 1100+ population-based datasets estimated 828 million adults (≥18 years) living with diabetes in 2022, up from ~200 million in 1990, with the steepest relative increases in low- and middle-income countries (LMICs) [5]. Consistent figures from the World Health Organization (WHO) report ~830 million people of all ages with diabetes in 2022 and highlight substantial treatment gaps—more than half were not taking glucose-lowering medication in 2022, with coverage lowest in LMICs [6]. Alternative surveillance systems using different age bands yield lower absolute counts but similar trends: the International Diabetes Federation (IDF) Diabetes Atlas 2025 estimates 590 million adults aged 20–79 years (11.1%; ~1 in 9) living with diabetes, with >40% undiagnosed, and projects ~853 million adults by 2050 [7]. The World Health Organization (WHO) reports that diabetes accounted for 1.6 million fatalities in 2019 and was the eighth most common cause of death [8] and accounted for at least USD 1 trillion in annual health expenditure [9]. Long-range forecasts from the Global Burden of Disease (GBD) collaboration and allied analyses suggest that, without intensified prevention and care, the number of people living with diabetes could exceed 1.3 billion by 2050, with the bulk of cases representing type 2 diabetes [10].

Clinically, diabetes ranges from an insidious, asymptomatic course to acute, symptomatic hyperglycemia. Across types, common symptoms include polyuria, polydipsia, polyphagia, unintentional weight loss, fatigue, blurred vision, slow-healing wounds, and recurrent infections [11,12]; however, many individuals—especially with T2D—may be asymptomatic for years and are diagnosed via opportunistic screening or evaluation for complications [13,14]. Symptom tempo often reflects etiology: type 1 diabetes (T1D) can evolve over weeks to months and may first present with diabetic ketoacidosis (DKA)—occurring at diagnosis in roughly one-quarter to one-third of children in North America—whereas T2D typically develops gradually, sometimes accompanied by signs of insulin resistance (e.g., acanthosis nigricans) and metabolic comorbidity [15,16,17].

Underlying pathophysiology differs by type but converges on hyperglycemia-mediated tissue injury. In T1D, immune-mediated β-cell destruction culminates in absolute insulin deficiency [18]; in T2D, peripheral insulin resistance (skeletal muscle, adipose tissue, liver) interacting with declining β-cell function produces relative insulin deficiency over time [19]. Other specific types encompass defined genetic defects of β-cell function (e.g., MODY) [20], exocrine pancreatic disease [21], endocrinopathies [22], and medication-induced hyperglycemia [23]. These distinctions matter clinically because they shape natural history, complication profiles, and therapeutic strategies summarized in the American Diabetes Association (ADA) Standards of Care 2025 [24].

Diabetes causes changes in brain function. Functional MRI studies in type 2 diabetes consistently show altered resting-state networks most notably disrupted default-mode network (DMN) connectivity that tracks with subtle cognitive deficits [25,26]. Task fMRI often reveals reduced dorsolateral prefrontal activation during memory/working-memory demands and blunted DMN deactivation, with some reports of compensatory hyperactivation in frontoparietal control networks [27,28,29]. Meta-analyses of resting-state measures (e.g., ALFF, ReHo) converge on regionally abnormal spontaneous activity across visual, sensory, and executive systems, supporting widespread but heterogeneous functional dysregulation [30,31]. These neural alterations are reproducible in newer multi-index rs-fMRI work, though their interpretation should consider diabetes-related differences in cerebrovascular reactivity that can modulate the BOLD signal [32,33].

**Figure 1:**
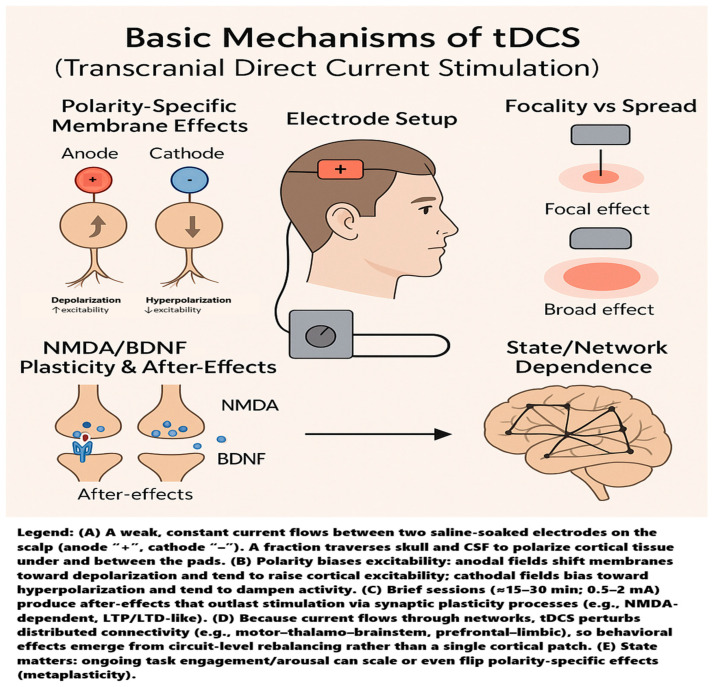
Basic mechanisms of tDCS.

Transcranial direct current stimulation (tDCS) is a noninvasive way to nudge brain activity by running a small, steady direct current across the scalp through two or more saline-soaked sponge electrodes—typically an anode and a cathode—positioned on the head [34]. The method is painless, well-tolerated, and safe, with only a few mild side effects reported [35]. A compact, battery-powered device delivers 0.5–2 mA of current, a fraction traversing the skull to influence cortical neurons. Polarity matters: anodal stimulation tends to depolarize neurons and raise their excitability, whereas cathodal stimulation usually hyperpolarizes them and dampens activity [36,37]. Because current flows between the pads, conventional tDCS affects a relatively broad area; using smaller electrodes can make the effect more focal [38]. Standard sessions that last 15–30 min to 20 min are common, producing physiological changes that outlast the stimulation. Even three minutes can leave detectable after-effects, and ten or more minutes at 1–2 mA can stabilize these changes for at least an hour [37,39]. A single 15 min session can modulate cortical excitability for roughly 90 min, with repeated sessions extending the duration of these effects [40,41,42]. These after-effects are thought to arise from subtle shifts in membrane polarization that bias synaptic plasticity, rather than from direct neuronal firing [43]. Indeed, multiple studies show that tDCS can induce long-lasting, LTP-like or LTD-like changes in the cortex, consistent with Hebbian-style learning [44]. This plasticity depends critically on NMDA receptor signaling and modulators such as BDNF; for example, pairing anodal polarization with low-frequency input can yield persistent LTP in rodent cortex, an effect abolished by NMDA antagonists [41]. Crucially, these mechanisms are context-dependent. The brain’s ongoing state-rest vs. task engagement, arousal level, even time of day, can shape, blunt, or sometimes reverse the expected polarity-specific outcomes (so-called metaplasticity) [45,46]. And tDCS does not act in isolation on a single cortical patch: altering one node (e.g., M1) perturbs activity across connected networks [47]. Neuroimaging confirms shifts in large-scale functional connectivity under tDCS, meaning behavioral effects emerge from the state of the broader network, not just the stimulated site [48,49]. The basic mechanisms of tDCS are illustrated in Figure 1.

tDCS is generally used in three broad ways. First, as a treatment adjunct for brain and mental health conditions—think depression [50], neurodegenerative diseases [51], schizophrenia [52], pain syndromes [53], and neurodevelopmental disorders [54]. Second, as a tool to study and boost cognition [55], with reports of benefits for language learning [56], numerical skills [57], and executive functions [58]. And third, in sports science, it has been explored to sharpen athletes’ psychophysical performance [59].

It was previously mentioned that diabetes is associated with impaired neurological function, including brain connectivity and excitability. This suggests that tDCS may be a viable option for alleviating various diabetes-related symptoms. This review aims to synthesize and analyze all studies using tDCS in patients with diabetes. We examine how this neuromodulation technique affects various disease and functional parameters and elucidate potential mechanisms of action. Furthermore, we broadened the scope of this work. We identified other studies that did not include patients with diabetes but examined the effects of tDCS on metabolic and physiological parameters relevant to diabetes. This approach allows for further exploration of the potential benefits of tDCS in alleviating the symptoms of this condition.

## 2. Materials and Methods

### 2.1. Data Sources and Search Strategy

Two reviewers (J.Ch. and D.K.) independently searched PubMed/MEDLINE, the Cochrane Library, Google Scholar, Scopus, and ResearchGate for studies on transcranial direct current stimulation (tDCS) in diabetes. Searches covered publications from 1 January 2008 through 31 August 2025; the final database access was August 2025. No language restrictions were applied. The exact Boolean string used in all databases was the following: ((“transcranial direct current stimulation” OR “tDCS”) AND (“diabetes” OR “diabetic”)).

The query was applied to title, abstract, and author-provided keywords where platform options allowed. No filters other than publication date were used at the search stage. All retrieved records from the three sources were exported and deduplicated before screening (automatic matching by title/author/year followed by manual verification).

### 2.2. Eligibility Criteria

We prespecified eligibility using PICOS. We included interventional human studies enrolling participants with diabetes (any type), in which tDCS was delivered as the study intervention, regardless of comparator (sham, active comparator, or single-arm pre–post designs). We accepted randomized and non-randomized trials and single-case interventional reports to reflect the included evidence. No restrictions were applied on language or clinical outcomes. We excluded non-interventional articles (reviews, editorials, letters), preclinical work, conference abstracts without full text, studies where tDCS effects could not be isolated from other neuromodulation, and studies without a diabetic cohort or without separable data for participants with diabetes. Two reviewers independently screened titles/abstracts and full texts with consensus adjudication. Search dates, sources, and the Boolean string are detailed in Section 2.1. Inclusion and exclusion criteria for study selection are presented in Table 1.

Inclusion criteria:

Study design: Clinical trials (randomized or non-randomized, parallel or crossover), case studies.

Population: Human participants with diabetes (any type, age, sex).

Intervention: tDCS as a standalone or adjunctive intervention.

Timeframe: Publications dated 1 January 2008–31 August 2025.

Language: Any (no restrictions).

Exclusion criteria:

Non-interventional designs (e.g., narrative reviews, systematic reviews, meta-analyses, editorials, letters, opinions).

Preclinical studies (animal or in vitro), methodological papers without patient data.

Conference abstracts without an accompanying full-text article.

Studies where tDCS was not part of the intervention, or mixed-modality neuromodulation, where the specific effects of tDCS could not be isolated.

Studies on non-diabetic populations or mixed cohorts without separable data for participants with diabetes.

### 2.3. Screening Process

Study selection followed a two-stage screening process conducted independently by both reviewers (J.Ch., D.K.). All decisions and reasons for exclusion at the full-text stage were recorded. Discrepancies were resolved by consensus.

#### 2.3.1. Title and Abstract Screening

After deduplication, titles and abstracts of all records were screened against the eligibility criteria. At this stage, a record was retained for full-text review if it potentially (i) enrolled participants with diabetes; (ii) evaluated tDCS as an intervention; and (iii) described a clinical trial design. Records clearly meeting exclusion criteria (e.g., reviews, animal studies, non-tDCS interventions) were excluded.

#### 2.3.2. Full-Text Assessment

Full texts of potentially eligible studies were retrieved and assessed against all predefined inclusion/exclusion criteria. Particular attention was paid to the following: (i) confirmation of a clinical-trial design in humans; (ii) the presence of tDCS as the intervention; (iii) the study population being individuals with diabetes (or a clearly separable diabetic subgroup); and (iv) the publication date between 1 January 2008 and 31 August 2025. Where information was ambiguous (e.g., unclear diagnosis, mixed interventions, or incomplete reporting), inclusion required that the criteria above were explicitly satisfied in the article.

## 3. Results

**Figure 2:**
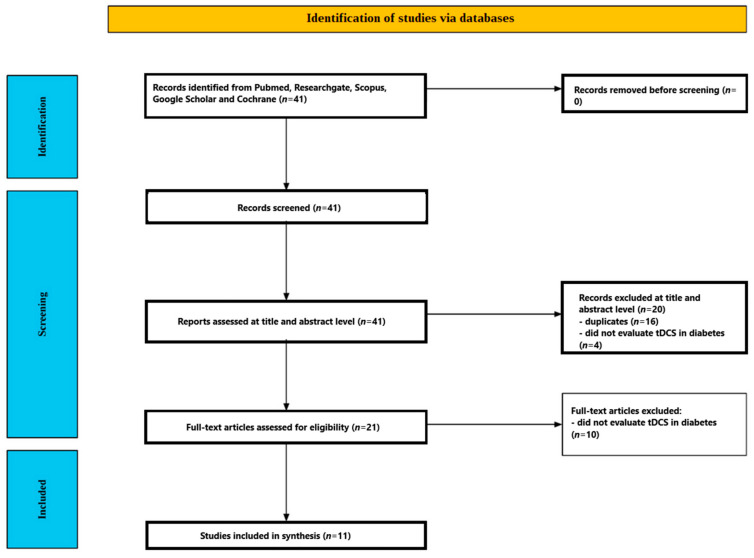
Study selection flow.

Figure 2 outlines the study selection flow. The database search yielded 41 records. After removing duplicates (n = 16) and excluding titles/abstracts that did not evaluate tDCS in people with diabetes (n = 4), 21 articles moved to full-text review. Of these, 10 were excluded because they did not assess the effect of tDCS on diabetic participants. Ultimately, 11 studies met the criteria and were included in the review [60,61,62,63,64,65,66,67,68,69,70]. The included studies are summarized in Table 2.

### 3.1. Participants’ Characteristics

Across the included reports, samples were composed primarily of adults with long-standing type 2 diabetes and clinically confirmed complications affecting pain, peripheral nerves, or vision. Most trials enrolled middle-aged to older adults (overall range 18–70 years; typical group means ~52–63 years) with diabetes duration ≥5 years, where stated [60,67,68,69]. Sex distribution was variably reported; in one four-arm trial, most participants were female [69], and another quality of life (QoL) study enrolled 12 women and 8 men [68]. When anthropometrics were provided, obesity was common (e.g., mean BMI 36.7 kg/m^2^ in [68]).

Neuropathic pain phenotypes predominated. Several studies required painful diabetic peripheral neuropathy (DPN/PDPN) with minimum pain severity (e.g., Visual Analog Scale (VAS) ≥ 4) and/or diagnostic thresholds (e.g., Douleur Neuropathique en 4 Questions (DN4) > 4; electrodiagnostic confirmation or Neuropathy Total Symptom Score (NTSS) > 6) and stipulated pain chronicity of at least 3 months [61,66,67,68]. Three companion reports analyzed the same randomized, four-arm cohort from the Bonab Diabetes Association (n = 48; block randomized to M1, F3, combined M1 + F3, or sham) with inclusion of type 2 diabetes ≥ 5 years and specialist-diagnosed neuropathic pain [60,69,70]. Other designs included a randomized comparison of tDCS vs. transcutaneous electrical nerve stimulation (TENS) in outpatient DPN [67], a phase-II pilot in diabetic polyneuropathy [63], and a single-patient case report (65-year-old male with plantar fasciitis and type 2 diabetes) [65]. One study targeted central visual dysfunction, enrolling patients with proliferative diabetic retinopathy (PDR) rather than neuropathic pain (n = 22; randomized 1:1 to cathodal V1 vs. sham) [62]. Another enrolled patients with severe DPN (Dyck 2a/2b) plus an age-/education-matched healthy control group for baseline cognitive comparisons (patients: n = 16; controls: n = 16) [64].

Cognitive status and psychiatric comorbidity were explicitly screened in several trials. One sham-controlled study required MMSE > 24 and excluded major depression and substance use disorders [61]; the Dyck-graded DPN sample demonstrated lower MoCA scores consistent with mild cognitive impairment at baseline relative to controls [64]. Trials commonly excluded unstable medical/psychiatric illness, implanted stimulators, other pain etiologies, and standard tDCS contraindications [61,63,66,67,68]. Concomitant analgesics were permitted if stable (e.g., pregabalin, gabapentin, NSAIDs), with no dose changes during the study [66]; the case report maintained existing medications during stimulation [65].

Baseline symptom severity was moderate to high, as quantified: Short-Form McGill Pain Questionnaire–Version 2 (SF-MPQ-2) totals ≈69–71 in the Bonab cohort [60], and mean VAS pain was around 5.7 in the PDPN randomized controlled trial (RCT) [66]. Groups were generally comparable at baseline on demographics and clinical variables when reported [62,66,69]. Attrition was low overall; one PDPN RCT reported 12 dropouts before completion (60/72 completing all sessions) with mild stimulation-related adverse effects (e.g., itching, headache) [66].

### 3.2. tDCS Interventions

Protocols varied by target, dose, schedule, and sham method, but most trials used conventional sponge–electrode tDCS at low intensity with standard 10–20 EEG positioning.

#### 3.2.1. Targets and Montages

Analgesic protocols predominantly targeted the primary motor cortex (M1), typically with the anode over C3 (left M1) and then a return over the contralateral supraorbital area (e.g., Fp2) [63,65,66,67,68]. Some designs sequentially stimulated left and right M1 within a session [60,69,70]. Cognitive protocols targeted the dorsolateral prefrontal cortex (DLPFC): anode at F3 with cathode at F4 (bilateral prefrontal) [61], or anode over the right DLPFC with an extracephalic return on the left cheek [64]. A vision-focused study delivered cathodal tDCS to the primary visual cortex (V1; Oz) with an extracephalic anode on the right shoulder for focality [62]. Combined-site protocols applied M1 and F3 within the same session (M1 + F3) [60,69,70].

#### 3.2.2. Intensity, Duration, and Session Structure

Most active arms used 2 mA with 20 min epochs per target [61,63,64,65,66,69,70]. Exceptions included 1 mA for 10 min (V1; single session) [62] and 1 mA for 20 min, three times weekly for two months in two outpatient programs [67,68]. Multi-site sessions in the four-arm Bonab trials lasted 40 min at 2 mA: M1-only (20 min left M1 then 20 min right M1), F3-only (40 min F3), and combined (10 min left M1, 10 min right M1, 20 min F3) [60,69]; a companion report described 10–20 min per site within the same 40 min framework [70]. Acute 5-day courses (2 mA, 20 min, consecutive weekdays) were used in PDPN analgesia [66], a phase-II polyneuropathy pilot [63], a plantar fasciitis case [65], and a DLPFC + working-memory protocol [61]. A crossover cognitive study used 2 mA for 15 min per session (active vs. sham days ≥ 24 h apart) [64].

#### 3.2.3. Electrodes and Materials

When specified, saline-soaked sponge electrodes measured 5 × 7 cm [61,62]; the V1 protocol reported a current density of ~0.029 mA/cm^2^ at 1 mA [62]. Device models included NeuroStim2 with two electrically isolated channels (enabling sequential site dosing within a session) in the Bonab trials [60,69,70] and Gymna Uniphy Phyaction 787 in the two-month outpatient program [68].

#### 3.2.4. Sham Procedures and Blinding

Sham conditions replicated the set-up and initial sensations, then discontinued the current: ~20–30 s of active stimulation (often with 30 s ramp up/down) before power-off were used to maintain blinding [60,61,62,63,64,66]. Trials were double- or triple-blind with concealed allocation where stated [60,61,63,66,69,70].

#### 3.2.5. Concomitant Activities and Co-Interventions

One study paired DLPFC tDCS with adaptive visuospatial N-back training throughout each 20 min session [61]. Several analgesia trials allowed stable background analgesics without dosage changes [65,66]. A comparative electrotherapy trial contrasted M1-targeted tDCS with TENS delivered to the painful territory; both were administered 20 min/session, three times weekly for two months [67]. Most other sessions were conducted at rest in a quiet setting [63].

#### 3.2.6. Dose Frequency and Total Exposure

Total exposure ranged from a single 10 min V1 session [62], through short 5-session courses (100–120 min total active stimulation) [61,63,65,66], to extended programs of ~24 sessions over two months at 1 mA (≈480 min total) [67,68] and intermediate 12-session courses at 2 mA over four weeks (480 min total, distributed across one to three targets per session) [60,69,70]. Across studies, protocols were well-tolerated, with typical transient sensations (itching/tingling; occasional headache) and rare discontinuations [63,66].

### 3.3. tDCS Results in People with Diabetes

tDCS produced domain-specific benefits across pain, cognition/affect, sleep/quality of life, and vision. Effects depended on target (e.g., M1 vs. DLPFC vs. V1), dose schedule, and whether stimulation was combined with behavioral training. Below, we synthesize outcomes by domain.

#### 3.3.1. Neuropathic and Musculoskeletal Pain

1. Motor Cortex (M1)-targeted Analgesia

Two randomized trials and several pre–post programs showed clinically meaningful pain reductions with anodal M1 protocols. In a four-arm, double-blind RC, SF-MPQ-2 scores fell after 12 sessions in all active arms but not in the sham arm. All active vs. sham were significant at posttest; M1 was superior to F3. At two-month follow-up, the reductions persisted, with each active arm better than sham (*p* < 0.001) but no differences among active arms [60].

A second RCT in PDPN compared five daily sessions over M1, DLPFC, or sham. VAS pain decreased ~34% in M1 vs. ~22% in DLPFC and ~14% in sham; benefits in M1 were maintained at 2 and 4 weeks. Notably, 65% in M1 achieved ≥30% pain reduction vs. 35% DLPFC and 5% sham. Pressure pain thresholds increased ~6.4% in M1; Clinical Global Impression (CGI) improved more in M1 than in DLPFC or sham. Adverse effects were mild (itching/headache); one dropout for headache [66].

2. Comparative Electrotherapy and Extended Low-Intensity Courses

Over two months, both tDCS (M1-targeted, 1 mA, 20 min, 3×/week) and TENS produced considerable and similar improvements on the Neuropathy Pain Scale (NPS); no between-group difference was noted [67]. A separate two-month pre–post study with the same 1 mA schedule reported significant NPS reductions across pain qualities, alongside broad Neuro-QoL gains (e.g., depression −64.63%, anxiety −45.71%, fatigue −46.47%) [68].

3. Functional Pain and Polyneuropathy Pilot

In a phase-II pilot (five sessions of anodal M1), 36-Item Short Form Health Survey (SF-36) bodily pain improved substantially, with concurrent gains in physical functioning and mobility markers (TUG, 6MWT); strength/flexibility did not change [63].

4. Case Evidence (Plantar Fasciitis with Diabetes)

A 65-year-old patient receiving five anodal M1 sessions improved on the VAS, reduced pain-related anxiety, and discontinued opioids by day 2; side effects were minimal [65].

5. Negative/neutral Pain Findings with DLPFC + Working memory training (WMT)

When tDCS (2 mA anode F3/cathode F4) was paired with adaptive N-back training for 5 days, pain (VAS), Self-reported Leeds Assessment of Neuropathic Symptoms and Signs (S-LANSS) and Neuropathy and Foot Ulcer–Specific Quality of Life (NEPIQOL) showed no significant time × group effects—active was not superior to sham [61].

6. Take-Home for Pain

Across controlled trials, M1 targeting delivered the most consistent and durable analgesia; DLPFC alone did not match M1 for pain relief in PDPN, though extended, lower-intensity community programs also yielded extensive pre- and post-improvements [60,63,66,67,68].

Across controlled pain trials, mean reductions on the VAS of 2–3 cm and ≥30% improvement are generally accepted as minimal clinically significant differences (MCIDs) for neuropathic pain. In the reviewed M1-tDCS studies, 65% of participants exceeded this threshold [66], and SF-MPQ-2 total score reductions of 7–8 points surpassed the MCID range (5–8) reported for chronic neuropathic conditions, supporting the observation that observed analgesia was clinically meaningful rather than statistically nominal [A1, A2].

#### 3.3.2. Cognitive, Psychiatric, and Affective Outcomes

1. Working Memory (WM)

Pairing DLPFC tDCS with concurrent WM training yielded selective WM benefits. Verbal 2-back performance (d′) improved from baseline to 1 month in the active group but not in the sham; visuospatial WM (Corsi forward span) also increased. No superiority for active was detected in semantic/phonemic/alternating fluency or CVLT immediate recall; delayed recall was unchanged [61].

In severe DPN (Dyck 2a/2b), a within-subject crossover (2 mA right DLPFC, 15 min) showed post-stimulation gains on a computerized Corsi task, especially under motor-interference load. Baseline nerve conduction velocity (NCV) correlated with the hardest-condition performance (worse NCV → lower span). Still, this association disappeared after active tDCS due to preferential improvement in low performers (baseline spans < 4) [64].

2. Anxiety, Depression, and Distress

In the DLPFC + WMT RCT, anxiety (Beck Anxiety Inventory, BAI) decreased from baseline to 1 month in the active group with no change in the sham; depression (BDI) did not improve. Pain outcomes were neutral in this study (see above) [61].

In the Bonab four-arm cohort (12 sessions; M1, F3, M1 + F3, sham), psychological distress (Depression, Anxiety, and Stress Scales, DASS-42) declined over time with significant main effects in time and group; all active groups improved more than sham, with no differences among active arms and benefits maintained at 1- and 3-month follow-ups [70].

#### 3.3.3. Sleep and Health-Related Quality of Life (HRQOL)

In a double-blind, four-arm RCT, sleep quality (Pittsburgh Sleep Quality Index, PSQI) and SF-36 composites improved over time in active arms, but only the combined M1 + F3 protocol separated from sham in post hoc tests for PSQI. For SF-36, the com-bined arm outperformed both sham and F3-only; M1-only did not differ significantly from sham or F3-only in reported pairwise comparisons. Improvements achieved by the end of 12 sessions persisted at 1 and 3 months (no differences among post-baseline timepoints) [69].

Converging evidence from a phase-II pilot showed larger HRQoL gains in the ac-tive M1 group vs. sham, concentrated in physical domains (physical functioning, bodi-ly pain, functional capacity), with parallel improvements in TUG and 6MWT. Mood-related domains were less responsive [63]. Pre–post community tDCS further suggest-ed broad Neuro-QoL benefits, though without a control arm, these estimates should be interpreted cautiously [68].

#### 3.3.4. Visual Function in PDR

A single-session, randomized sham-controlled study applying cathodal tDCS to V1 (Oz; 1 mA, 10 min) improved visual acuity (logarithm of the minimum angle of resolution, LogMAR) in both eyes of the active group with no change in sham. Number acuity reaction times improved substantially in the active group, while accuracy re-mained at ceiling; sham was unchanged. Authors interpret this as reduced cortical “neural noise” enhancing signal-to-noise and downstream visual processing efficiency [62].

### 3.4. Safety

No serious adverse events, skin burns, or discontinuations due to adverse effects were reported.

### 3.5. Risk of Bias

The RoB2-based risk assessment of bias in the included studies is presented in Table 3.

## 4. Discussion

Across heterogeneous diabetic populations and outcomes, a consistent pattern emerges: M1-targeted tDCS most reliably improves pain and function, DLPFC-targeted tDCS selectively benefits cognition and anxiety, and combining cortical targets (M1 + DLPFC) broadens the clinical footprint to sleep and quality of life domains. These modality- and site-specific effects align with what is known about tDCS network physiology and the pain neuromatrix, where sensorimotor, prefrontal, thalamic, and salience circuits contribute differentially to nociception, affect, vigilance, and sleep–wake regulation. The single-case plantar fasciitis report underscores the speed and tolerability with which motor cortex protocols can de-escalate severe peripheral pain when drugs fail. The reviewed trials argue that tDCS is not a one-size-fits-all tool; instead, target choice should be matched to the dominant clinical phenotype (e.g., pain vs. anxiety/cognitive burden vs. sleep disruption), and multi-site protocols may be preferable when goals are multidimensional. A graphical summary illustrating the main domains potentially improved by tDCS in diabetes is presented in Figure 2.

### 4.1. Mechanistic Interpretation

Diabetes-related changes in energy homeostasis are coordinated by cortico-subcortical loops that prominently include the prefrontal cortex and the hypothalamus, the latter integrating nutrient sensing with autonomic and endocrine outputs [71,72,73]. Prefrontal dysregulation in diabetes, evident in task and resting-state fMRI, co-occurs with impaired hypothalamic control of energy balance and sympathetic tone, contributing to insulin resistance and glycemic instability [74,75,76,77]. tDCS can interact with these nodes by biasing large-scale cortico-subcortical signaling and shifting neurotransmitter tone, including dopaminergic gain relevant to reward-driven intake and executive control [78]. At the same time, NMDA-dependent plasticity may support durable state changes. In parallel, attenuation of stress-axis signaling could reduce hypothalamic insulin-antagonizing drive. These pathways provide a biologically coherent account of how weak-current prefrontal and network-targeted stimulation might influence ingestive behavior and neuro-metabolic coupling beyond local excitability effects.

#### 4.1.1. Why M1 for Analgesia?

M1 is analgesic not because it “moves muscles”, but because it tops a cortico–thalamo–brainstem axis that modulates nociceptive gain. M1 projects to thalamic relays and to hubs of the descending pain system—the periaqueductal gray (PAG) and rostroventromedial medulla (RVM)—which gate dorsal horn transmission. Contemporary models show PAG→RVM output regulates spinal nociception via serotonergic/opioidergic mechanisms and that cortical drive can bias this gate anti-nociceptively—the pathway motor cortex stimulation is positioned to recruit [79,80].

Clinically, converging noninvasive and invasive neuromodulation data highlight M1’s privileged role. Evidence-based rTMS guidelines repeatedly identify M1 as the most reliable cortical site for analgesia across neuropathic and nociplastic pain—an observation commonly extrapolated to weak-current stimulation of the same network with tDCS. Mechanistic reviews likewise frame M1-tDCS as a “top-down” lever via connectivity with the thalamus, cingulo-insular salience regions, and brainstem pain circuits [81,82].

Human imaging links M1-tDCS to subcortical reweighting: in volunteers, anodal M1-tDCS increases M1–thalamus coupling and alters cortico-striatal interactions; in fibromyalgia, multi-session M1-tDCS changes resting-state connectivity among the thalamus, sensorimotor cortex, posterior insula, PAG, and medial prefrontal cortex, with greater pain relief tracking larger reductions in thalamo–sensorimotor and thalamo–insula coupling [83,84,85].

Molecular imaging shows recruitment of endogenous μ-opioid mechanisms: PET demonstrates increased μ-opioid neurotransmission during a single M1-tES session in regions including the PAG and thalamus [86], and high-definition M1-tDCS in orofacial pain relates symptom improvement to μ-opioid receptor dynamics [87].

At the cortical input stage, weak currents over the sensorimotor cortex modulate somatosensory-evoked responses, consistent with transient dampening of early cortical representations of peripheral input—a corticocortical gating that complements brainstem routes [88].

Psychophysically, meta-analyses across chronic pain show that active tDCS—most often over M1—reduces clinical pain vs. sham, including in remote/home delivery, with clinically meaningful end-of-treatment effects. Experimental threshold results are heterogeneous: some syntheses find no reliable change, others report increases in pressure/heat pain thresholds and pressure pain tolerance after multi-session courses. This pattern fits a model in which M1-tDCS chiefly recalibrates network-level valuation/amplification of nociceptive input, with thresholds secondarily affected depending on dose, montage, and baseline state [89,90,91,92,93].

**Figure 3:**
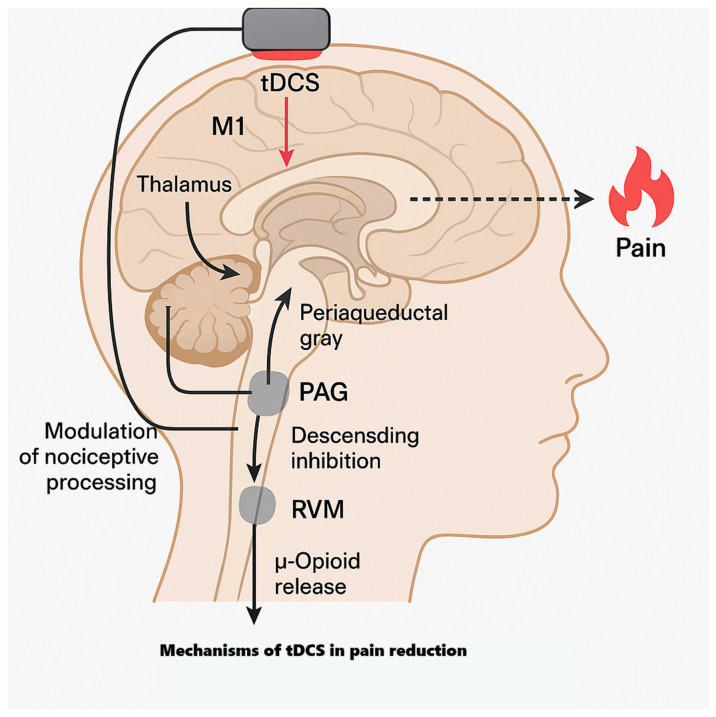
Mechanisms of tDCS in pain.

In sum, facilitatory M1 currents bias cortical output, retune corticothalamic/striatal coupling, recruit PAG/RVM-mediated descending inhibition (with demonstrable μ-opioid engagement), and gate early somatosensory responses—yielding less coherent pronociceptive activity and lower pain after adequately dosed, repeated sessions. This system’s view explains M1’s repeated outperformance of other single cortical targets and why protocol parameters (electrode size/placement, current density, session number/spacing) determine durability. Related molecular and circuit pathways for diabetes-related pain are summarized in Figure 3.

#### 4.1.2. Why DLPFC for Anxiety and Working Memory?

The DLPFC sits atop the executive-control network and exerts top-down control over limbic generators of threat and arousal. Anxiety reflects dysregulated prefrontal–amygdala coupling—hyperreactive amygdala with insufficient prefrontal control—whereas effective regulation recruits lateral/medial PFC to dampen limbic output [94]. Causally, a randomized, double-masked fMRI study in high trait-anxious adults showed that a single prefrontal tDCS session acutely reduced bilateral amygdala threat reactivity and increased cortical markers of attentional control vs. sham [95]. Broader circuit syntheses likewise emphasize prefrontal control over fear acquisition/expression, and resting-state effective-connectivity work in GAD highlights failed top-down PFC→amygdala control as a system-level signature of symptom severity [96,97]. Clinically, meta-analyses indicate that prefrontal noninvasive stimulation can reduce anxiety, but with heterogeneous magnitude and durability tied to montage, dose, diagnosis, and task pairing [98]; 2024 syntheses report overall benefits of tDCS on general anxiety with variable long-term effects and promising yet heterogeneous reductions with left-DLPFC tDCS [99,100].

The same control-network logic underpins DLPFC’s role in working memory (WM). DLPFC supports maintenance/manipulation under interference [101,102], with local inhibitory/excitatory balance tracking WM capacity [103]. MRS shows that baseline DLPFC GABA predicts individual WM load capacity, and prefrontal tDCS can transiently shift cortical neurochemistry (e.g., increased Glx in stimulated left DLPFC), a plausible substrate for state-dependent enhancement [104,105]. Behaviorally, single-session studies in healthy participants yield small or null average WM effects, implying that stimulation alone is rarely sufficient [106]. Pairing tDCS with adaptive WM training changes the picture: randomized and systematic reviews show more reliable, durable gains on trained tasks and selective near-transfer, especially with online (during-training) stimulation [107]. Landmark experiments and recent quantitative syntheses (2022–2024) confirm that online left-DLPFC anodal tDCS steepens training curves and near-transfer, but overall, WM effects remain modest and parameter-sensitive—consistent with stimulation as a plasticity facilitator that needs the right task context [108,109,110,111].

Two practical levers sharpen outcomes for anxiety and WM. First, timing/dose: online > offline for skill acquisition, and dose–response is nonlinear, warranting careful tuning of current, duration, and repetition [112]. Second, task coupling: pair DLPFC tDCS with attentional-control tasks for anxiety (e.g., threat-distraction, reappraisal) or with adaptive updating/interference challenges for WM (e.g., n-back with distractors) to exploit state-dependent plasticity in the fronto-parietal network and its projections [113,114,115,116,117,118]. In practice, multi-session (≈10–20) courses of left- (often verbal WM/anxiety) or right-hemisphere (visuospatial WM) DLPFC stimulation delivered during well-designed training, with monitoring of near- vs. far-transfer and montage adjustments (e.g., bifrontal) when broader network engagement or asymmetric metabolite/connectivity readouts suggest it, are most likely to yield durable benefits. In short, DLPFC is a rational target when anxiety and executive deficits dominate: stimulation can acutely normalize amygdala reactivity and improve control-network efficiency, but robust, lasting gains usually require function-matched tasks and optimized dose/montage.

#### 4.1.3. Visual Cortex “Denoising” and Diabetic Retinopathy

Severe retinal disease (e.g., proliferative retinopathy) degrades afferent drive to the early visual cortex, yielding maladaptive hyperexcitability and aberrant spontaneous occipital activity; intrinsic activity becomes noisier, resting connectivity with higher-tier visual areas shifts, and, in extremes, release phenomena (Charles Bonnet) can occur [119]. Resting-state MRI in vision loss—including diabetic retinopathy—shows disrupted V1–extrastriate coupling, consistent with a “mis-tuned” network and motivating SNR restoration at early sensory stages rather than treating the retina and cortex in isolation [120,121,122].

Occipital tDCS modulates V1 excitability in a polarity-specific manner: anodal lowers phosphene thresholds and increases VEPs/contrast sensitivity, whereas cathodal raises thresholds and decreases VEPs, with replication across paradigms and montage-dependent nuances. The core result—cathodal dampening of spontaneous and evoked occipital responses—offers a plausible cortical “denoising” mechanism when retinal input is degraded [123,124,125,126,127].

This aligns with broader tES theory: performance can improve by adding noise to underpowered systems (stochastic resonance tRNS) or reducing endogenous noise in hyperexcitable systems (cathodal tDCS), with direction set by baseline state and task. In vision, tRNS boosts contrast detection and perceptual learning when signals are weak; after deafferentation, reducing cortical noise should likewise aid read-out—both fitting state-dependent plasticity models in the adult visual cortex [128,129,130,131].

Accordingly, better basic acuity and faster numerosity decisions after occipital cathodal stimulation are neurocomputationally plausible. Numerosity is represented from early visual cortex to posterior parietal maps; high-field fMRI shows topographic tuning. Cleaner V1 outputs enable more efficient downstream evidence accumulation, shortening reaction times without necessarily changing asymptotic accuracy, especially under ceiling performance [132,133,134].

A complementary mechanism is homeostatic rebalancing: adult visual cortex scales synaptic gain to stabilize activity. In persistently overactive networks, repeated cathodal currents can bias local inhibitory–excitatory balance toward stability, improving encoding of luminance, contrast, and spatial statistics underlying optotype recognition and dot-array numerosity—consistent with polarity effects depending on baseline excitability and montage [123,130].

Two practical points follow. Dose/montage matter: effects vary with electrode placement (e.g., Oz–Cz vs. extracephalic returns) that alter field direction through calcarine banks and laminar balance. Task context matters: engaging vision (acuity, crowding-limited reading, numerosity) during/soon after stimulation enhances state-dependent plasticity, as shown in perceptual-learning studies pairing stimulation with training [125,126,135].

Safety at the occipital site is favorable: reviews across thousands of sessions report mostly mild, transient sensations (itch/tingle), rare headache, no severe tissue injury within standard parameters, and typically benign, transient phosphenes—supporting short, repeated occipital sessions as an adjunct to enhance residual vision rather than replace retinal therapies [136,137,138].

#### 4.1.4. Why Multi-Site (M1 + DLPFC) Can Look Best on Sleep and QoL

A dual-target strategy is biologically sensible: it couples an analgesic lever (M1 → descending pain modulation) with an arousal/affect-control lever (DLPFC → fronto-limbic regulation) [80,139,140,141,142]. Anodal M1 engages the PAG–RVM axis and retunes thalamo-sensorimotor coupling, yielding meaningful analgesia across chronic pain conditions [83]; DLPFC stimulation reduces limbic threat responsivity and strengthens top-down attentional control, lowering hyperarousal and worry that prolong sleep latency and fragment sleep [95,143,144,145]. Nudging both pathways in one course reduces night pain and cortical arousal, typically producing larger gains in sleep quality and QoL than either pathway alone.

The pain–sleep link is robust and bidirectional: poor sleep heightens next-day pain sensitivity, while ongoing pain disrupts sleep continuity. Reviews and longitudinal studies show that sleep quality more reliably forecasts next-day pain than the reverse, explaining why analgesia without arousal control leaves residual sleep deficits and QoL drag [146,147,148,149]. Multi-site stimulation targets both halves of this loop.

Empirically, each node contributes domain-specific benefits. Randomized tDCS trials in fibromyalgia and other persistent pain states show M1 stimulation reduces clinical pain and improves global impact/QoL—often more durably after 10–20 sessions—and enhances sleep parameters [140,150]. Prefrontal tDCS (left DLPFC/dorsomedial) improves sleep quality in insomnia and mood–insomnia comorbidity on PSQI and polysomnography; HD-tDCS over midline prefrontal sites also improves sleep-onset latency and efficiency [151,152]. Combining analgesic and arousal-control targets therefore justifies expecting broader sleep/QoL benefits.

There is a nuance: in classic fibromyalgia work with five daily sessions, M1 increased sleep efficiency and reduced arousals, whereas the particular DLPFC montage used lengthened latency and reduced efficiency. Later insomnia and depression–insomnia studies—with different montages/doses and more sessions—found that prefrontal tDCS improves subjective and objective sleep. Differences likely reflect diagnosis, baseline arousal, montage (bifrontal vs. extracephalic; DLPFC vs. dorsomedial PFC), and dose. Practically, pairing M1 with a sleep-promoting prefrontal montage (optimized DLPFC/DM-PFC, more sessions, online stimulation during relaxation or CBT-I components) captures M1’s analgesic sleep benefits while adding prefrontal gains in hyperarousal control, worry, and mood—netting a larger PSQI/QoL signal [150,151,152].

QoL—spanning pain interference, role function, vitality, and emotional participation—tracks multimodal change. Trials adding tDCS to behavioral rehab (exercise, pain neuroscience education, CBT elements) yield superior improvements in pain-related disability and QoL vs. rehab alone, with home-based protocols enabling higher dose densities that predict durability. A multi-site plan “bakes in” the same logic neurally: M1 reduces sensory-discriminative pain and interference and DLPFC reduces cognitive–emotional costs (rumination, catastrophizing, threat reactivity), together lifting composite QoL scores more than either target alone [153,154].

**Figure 4:**
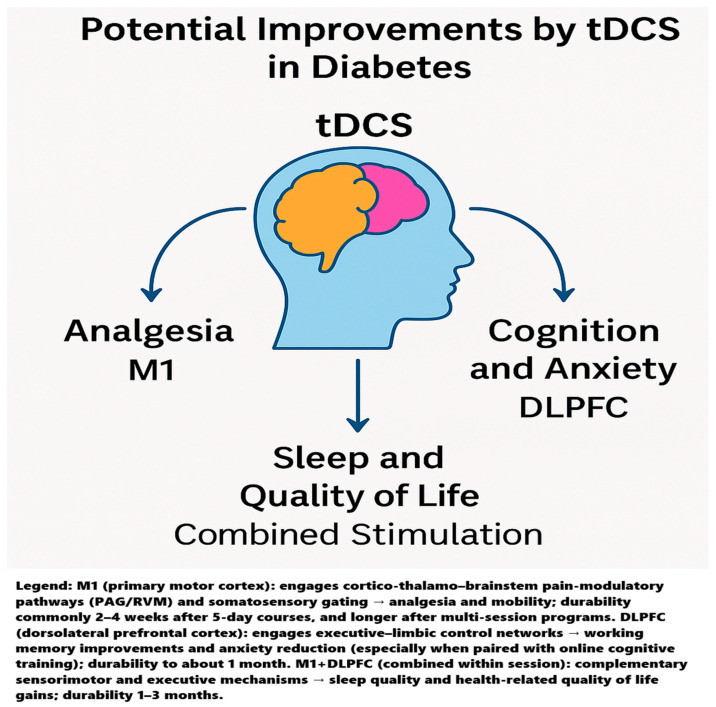
Graphical summary illustrating the main domains potentially improved by tDCS in diabetes.

In short, M1 + DLPFC aligns with the system’s biology of sleep and QoL: sleep stabilizes when nociceptive drive and cortical hyperarousal are both attenuated, and QoL rises when physical and emotional domains improve simultaneously—the integrative rationale behind dual-target protocols outperforming single-site approaches. Potential improvements by tDCS in diabetes is presented in Figure 4.

### 4.2. Durability of tDCS Effects

Follow-up durations ranged from immediate post-treatment to 3 months. Notably, pain, sleep, and psychological distress benefits persisted at 1–3 months in the four-arm Bonab trials [60,69,70], while shorter M1 courses maintained analgesia for 2–4 weeks [66]. These durability data, although limited, indicate short- to medium-term persistence of benefits consistent with other chronic pain tDCS literature.

### 4.3. Methodological Constraints and Interpretive Fragility

**Figure 5:**
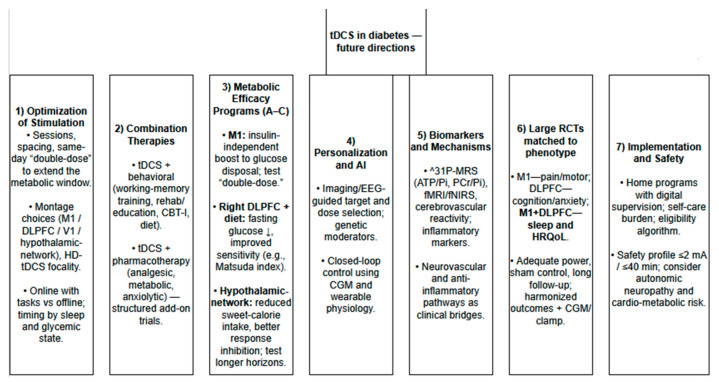
Future directions for research on tDCS in diabetes.

Future directions for research on tDCS in diabetes are outlined in Figure 5.

Across trials, the interpretability of the observed clinical signals is limited by deep methodological variability rather than mere noise. Protocols diverge on mechanistically non-equivalent dimensions (target selection, montage geometry, current density, exposure dose, online vs. offline delivery, and task-coupling), which plausibly alter effect direction and magnitude. The contrast between null pain effects under DLPFC-only online WM protocols and positive effects under M1 analgesic targeting exemplifies this sensitivity. Most studies are feasibility-scale (typical n ≈ 10–15/arm), increasing both false negatives and unstable positives; concomitant medications, unmeasured state variables (sleep, glycemia, arousal/time-of-day), and short follow-ups introduce confounding at the same order of magnitude as the expected tDCS effect. Heterogeneity in stimulation regimes parallels the metabolic domain, where montage, dose, repetition, and timing yield divergent neurometabolic profiles. Collectively, the current corpus signals biological plausibility but does not yet yield an exportable clinical recipe; adequately powered, phenotype-matched, mechanistically anchored, sham-controlled trials are required before any specific protocol can be considered promising rather than merely non-negative.

### 4.4. Clinical Implementation Barriers and Safety Contexts

Translating tDCS from controlled trials to routine diabetes care presents non-trivial implementation barriers. First, sustained use requires clinic-based delivery or home devices with competency training, remote verification of correct montage, and behavioral scaffolding to prevent attrition; adherence in other chronic neuromodulation programs declines steeply after week 4 without structured follow-up or digital supervision. Second, the feasibility of home-based tDCS in diabetes must be interpreted in light of competing self-management demands (diet, glucose monitoring, polypharmacy), meaning any tDCS program would add cognitive and organizational load to already saturated routines. Third, while tDCS is regarded as low-risk, diabetes cohorts are multimorbid: autonomic neuropathy, cardiovascular disease, arrhythmias, and dysautonomia can plausibly modulate hemodynamic responses to stimulation; uncontrolled hyperglycemia and labile blood pressure may alter cerebral reactivity and increase variability of response. Reported adverse events in trials were mild, but exclusion windows (unstable disease, acute decompensation, implanted devices) were expansive; thus, observed safety reflects selected—not real-world—populations. Therefore, a clinically realistic deployment would require stepwise screening, supervised initiation with remote compliance monitoring, and integration into existing care pathways rather than standalone adoption.

### 4.5. Toward Neuroimaging-Guided Personalization Instead of One Size Fits All

Emerging neuroimaging approaches provide a path away from generic montage choices toward phenotype-resolved dosing. Resting-state fMRI can quantify connectivity within pain–salience and executive networks; in diabetes, reproducible alterations (DMN dysconnectivity, blunted DLPFC task-engagement, thalamo-sensorimotor coupling shifts) offer stratification variables that could pre-select individuals more likely to respond to M1-anchored vs. DLPFC-anchored stimulation. EEG markers (frontal midline theta, high-beta asymmetry, pain-related evoked potentials) can serve as fast, low-cost signatures of cortical “state” to time stimulation windows or titrate current density online. In metabolic phenotypes (metabolic syndrome, obesity–diabetes overlap, autonomic neuropathy), neuro-metabolic imaging (^31^P-MRS, BOLD reactivity of hypothalamic networks) could select patients who express insulin-independent disposal shifts vs. those in whom the behavioral–executive route (DLPFC) dominates. Such stratification enables not only cognitive–affective matching (pain/anxiety/sleep-dominant subtypes) but also true dose-geometry personalization (montage selection, field orientation, current density, and task-coupling) based on objective neural or metabolic profile rather than a universal protocol.

### 4.6. Genetic Moderators of tDCS Responsiveness

Inter-individual variability in tDCS effects likely reflects, in part, genetic modulation of synaptic plasticity and neuromodulatory tone. Because canonical after-effects depend on NMDA-receptor signaling and are facilitated by BDNF-mediated plasticity, functional variants within glutamatergic and neurotrophin pathways could shift dose–response curves and durability (e.g., by altering thresholds for LTP/LTD-like changes). Dopaminergic and GABAergic genes that tune cortical gain and network inhibition may further bias montage-specific outcomes (executive-control vs. sensorimotor targets). In diabetes-spectrum phenotypes, where stress-axis and neuroenergetic responses to stimulation vary, genetic moderators might also help explain who expresses insulin-independent glucose disposal or neurometabolic signatures after anodal dosing. Pragmatically, genetics should be considered hypothesis-generating covariates for stratification and mediation analyses in future trials rather than near-term selection tools; combining genotype with state and circuit readouts (EEG/fMRI/^31^P-MRS) would offer the most informative personalization framework.

### 4.7. Brain–Gut Axis as a Plausible Downstream Pathway

tDCS may not act exclusively through central control of pain, affect, or executive function. Still, they could secondarily influence the brain–gut axis, which is increasingly recognized as a determinant of insulin sensitivity, low-grade inflammation, and metabolic homeostasis. Cortico–hypothalamic modulation can shift vagal efferent tone and stress-axis output, which regulate gut permeability, microbiota composition, and incretin signaling. In parallel, prefrontal control over reward valuation may alter dietary exposures that reshape microbiota and their metabolite profile (e.g., SCFAs), closing a bidirectional loop in which central neuromodulation and peripheral microbial ecology co-determine metabolic state. Although none of the reviewed trials incorporated microbiome endpoints, the convergence of tDCS effects on stress physiology, ingestive behavior, and autonomic output provides a mechanistic rationale to integrate gut-axis readouts into next-generation protocols, especially in phenotypes with metabolic syndrome, visceral adiposity, or inflammatory signatures.

### 4.8. AI-Enabled Adaptive Dosing and Closed-Loop Personalization

Beyond static protocol design, artificial intelligence enables dynamic rather than fixed neuromodulation. Continuous data streams from CGM, wearables (sleep, HRV, activity), and home devices can be fused by AI models to detect metabolic/physiologic states that either potentiate or blunt tDCS efficacy. In such a closed-loop paradigm, stimulation is not scheduled by the calendar but triggered or withheld based on real-time probability of benefit (e.g., pre-hyperglycemic drift, high stress-load/HRV collapse, short-sleep days). Parallel AI tools could extract latent phenotypes from fMRI/EEG/^31^P-MRS or clinical trajectories to recommend montage, current, and timing as a function of patient-state rather than population averages. This shifts tDCS from a fixed-dose, one-size-fits-all adjunct toward an adaptive, state-contingent intervention embedded in existing diabetes self-management infrastructure.

### 4.9. Neurovascular Coupling as a Candidate Translational Bridge

tDCS alters cerebral hemodynamics in a polarity- and state-dependent manner. fNIRS, ASL-MRI, and BOLD studies consistently show that anodal stimulation can increase regional cerebral blood flow (rCBF) and modulate neurovascular coupling, whereas cathodal stimulation tends to reduce it [155,156,157]. In diabetes and metabolic syndrome—where cerebrovascular reactivity and microvascular tone are impaired—such shifts could be clinically consequential: improved rCBF in prefrontal or sensorimotor hubs may secondarily improve substrate delivery (glucose, oxygen) to energetically stressed circuits, potentially aligning with the insulin-independent increases in glucose disposal observed in clamp-based experiments [158,159]. Because endothelial dysfunction, autonomic dysregulation, and inflammation co-drive both vascular and metabolic risk in diabetes, a neurovascular route provides a plausible explanatory bridge between weak-current cortical input and peripheral metabolic outcomes. It justifies adding vascular readouts (ASL-MRI, fNIRS, CVR testing) to next-generation trials.

### 4.10. Neuroinflammation as a Molecular Convergence Point for Metabolic and Cognitive Benefit

Low-grade neuroinflammation is now recognized as a mechanistic bridge between metabolic disease and cognitive dysfunction: microglial priming, cytokine spillover, and disrupted glia–neuron metabolic coupling contribute to insulin resistance, network dysconnectivity, and executive decline in diabetes [160,161]. tDCS interacts with this axis through circuit-level effects and via molecular gates: preclinical and translational data show reductions in pro-inflammatory cytokine expression, modulation of microglial reactivity, and restoration of synaptic plasticity under inflammatory load [162,163,164]. Given that metabolic syndrome and diabetes amplify inflammatory tone centrally and peripherally, the anti-inflammatory route provides a plausible unifying pathway linking neuromodulation to both metabolic and cognitive gains. Critically, current tDCS trials in diabetes did not include inflammatory biomarkers, meaning that an inflammation-linked benefit remains invisible to outcome adjudication; embedding inflammatory readouts in future trials is required to test whether tDCS protects the brain against inflammation-driven metabolic and cognitive degradation.

## 5. Future Perspectives on Using tDCS for Diabetes Management

**Figure 6:**
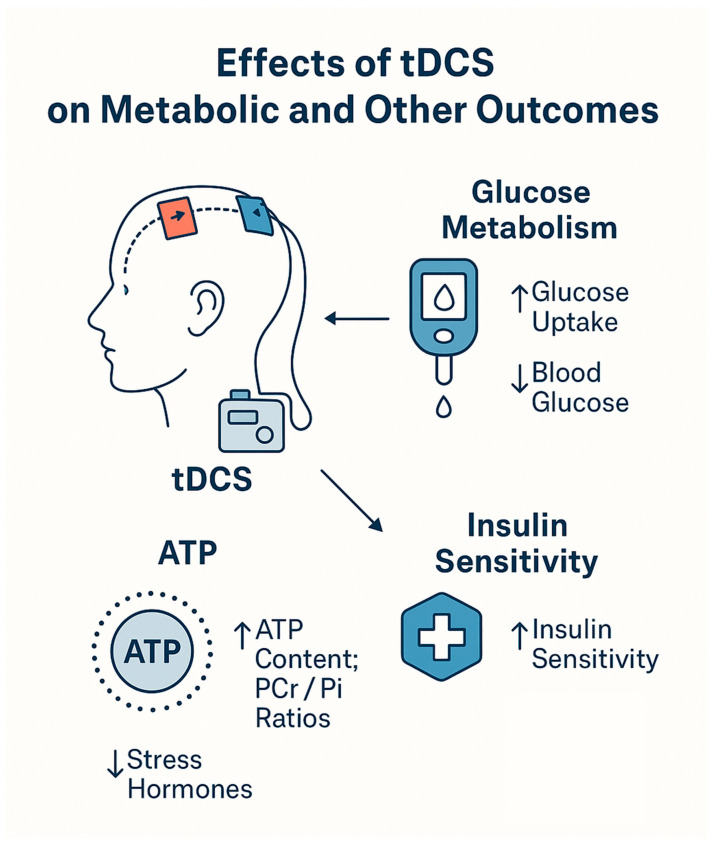
Metabolic and neurochemical mechanisms of tDCS.

We have discussed in detail the effects of tDCS on various parameters of pathophysiology and functioning in people with diabetes. However, other studies are using tDCS that did not examine the effects of tDCS on diabetics but instead examined the effects of tDCS on metabolic and neurochemical parameters relevant to the pathophysiology of diabetes. Six such studies were found. These studies were not included in the tDCS studies in diabetic patients, as they involve mostly healthy individuals. They provide background on existing findings, which further underscore the potential of tDCS to alleviate the effects of diabetes. These additional mechanisms are illustrated in Figure 6, and a description of these studies is presented in Table 4.

There is a consistent picture that emerges: brief, noninvasive increases in cortical excitability shift the brain’s high-energy phosphate balance (ATP, PCr) and, in parallel, alter systemic glucose handling largely without changing circulating insulin. In single-session, clamp-based experiments with anodal M1 tDCS, whole-body glucose infusion rates (a proxy for glucose disposal) rose above sham despite stable plasma glucose and insulin; ^31^P-MRS showed a characteristic ATP/Pi and PCr/Pi dip–rebound sequence that correlated with glucose uptake, and cortisol/ACTH and blood pressure fell [165]. A two-stimulation (“double”) protocol reproduced higher clamp M values and contemporaneous elevations in ATP/Pi and PCr/Pi, with a later cortisol reduction [167]. With eight daily sessions, post-stimulation glucose decreased on day 1, and the effect persisted and lengthened by day 8—again, insulin-independent—while neurometabolic responses adapted (ATP/PCr increases on day 1 absent by day 8) and higher PCr predicted lower glucose on day 8 [166]. Moving beyond healthy volunteers, right-DLPFC tDCS plus a hypocaloric diet produced greater reductions in fasting glucose. It improved Matsuda’s insulin sensitivity than diet alone, without changes in post-prandial AUCs or β-cell indices [168,169]. Finally, network-targeted (hypothalamus-anchored) tDCS improved inhibitory control and selectively reduced sweet-calorie intake, yet showed no change in OGTT-derived insulin sensitivity ~20 h later [170].

### 5.1. Mechanistic Fit with Diabetes Pathophysiology

#### 5.1.1. Neuroenergetic Trigger → Insulin-Independent Glucose Control

The ^31^P-MRS signatures after anodal stimulation-initial ATP/Pi and PCr/Pi decreases followed by recoveries above sham support a transient cerebral energy shortfall that can engage hypothalamic energy sensors (e.g., K_ATP channels). The observation that systemic glucose disposal rises without higher insulin during the clamp [165,167] is precisely the phenotype anticipated when the brain increases glucose effectiveness (insulin-independent uptake and/or suppression of hepatic glucose output). The positive coupling between ATP or PCr and glucose uptake [165] and the inverse PCr–glucose relationship after repeated dosing [166] tighten this brain–body link, suggesting that restoration of high-energy phosphate stores may index a pro-disposal state.

#### 5.1.2. Stress-Axis Modulation as a Co-Determinant of Insulin Resistance

Acute reductions in cortisol (and ACTH in one study) after tDCS ([165], replicated after the second session in [167]) point to a plausible route by which stimulation could reduce hepatic glucose production and improve peripheral insulin action, particularly in stress-sensitive hyperglycemia. The absence of systematic hormone changes in the 8-day study [166] cautions that stress-axis effects may be dose-, montage-, or context-dependent.

#### 5.1.3. Autonomic/Hemodynamic Context

The blood pressure reductions observed alongside metabolic benefits [165] are consistent with a central autonomic shift. Because autonomic dysfunction aggravates glycemic volatility and cardiovascular risk in T2D, these acute hemodynamic changes—if reproduced in diabetes—could compound metabolic gains.

#### 5.1.4. Executive Control and Ingestive Behavior

In obesity—a key driver of insulin resistance—right-DLPFC stimulation improved fasting glycemia and insulin sensitivity [168,169], while hypothalamus-network stimulation enhanced response inhibition and lowered sweet-calorie intake without short-term metabolic change [170]. Taken together, these results support a two-pathway model for translation: (i) direct neuro-metabolic modulation (M1 and possibly network-level targets) and (ii) behavioral control over hedonic intake (DLPFC, hypothalamus-network) that likely needs longer dosing and follow-up to manifest as glycemic improvement.

### 5.2. What the Dosing and Time Courses Imply

Across the six studies, three variables—montage/current, number and spacing of sessions, and time after stimulation—determine the metabolic signature with remarkable regularity. In single, 20 min, 1 mA anodal M1 sessions under clamp conditions, the systemic effect on glucose disposal is time-structured and biphasic: during the first ~30 min after stimulation begins, glucose infusion rate (GIR) trends downward vs. sham (*p* = 0.077), then reverses to a significant increase that persists for ~60 min (*p* = 0.012; overall treatment effect *p* = 0.031), with the maximum GIR elevation at ~200 min from stimulation onset before tapering; total glucose taken up over the experiment is higher than sham (*p* = 0.001) despite unchanged plasma glucose and insulin [165]. The cerebral energetic trajectory measured by ^31^P-MRS is synchronized but not identical in timing: ATP/Pi and PCr/Pi fall first, indicating an immediate energy draw, then overshoot above sham before returning toward baseline. For PCr/Pi, the drop reaches significance ~65 min after the end of stimulation (*p* = 0.021), followed by a significant rise (*p* = 0.005) and finally normalization (*p* = 0.975); ATP/Pi shows a sharp initial fall (*p* = 0.001), then a significant rise (*p* = 0.007) and later normalization (*p* = 0.775). Higher ATP and higher PCr/Pi correlate significantly with greater systemic glucose uptake (r = 0.549, *p* = 0.034; r = 0.552, *p* = 0.033), anchoring the clinical window to a neurometabolic readout [165]. Superimposed on this, cortisol and ACTH fall vs. sham (both interaction *p* = 0.004) and blood pressure decreases (systolic *p* = 0.021; diastolic *p* = 0.013), suggesting a concurrent autonomic–endocrine milieu that would be expected to favor insulin sensitivity [165].

The metabolic window can be reopened and extended when the same 1 mA, 20 min M1 dose is repeated within a session. In a protocol delivering a second identical stimulation 115 min after the first, GIR again increases for ~60 min after each stimulation (first stimulation: treatment effect F_1,14_ = 4.791, *p* = 0.042; second stimulation: interaction F_4.152,58.121_ = 3.451, *p* = 0.013). ^31^P-MRS shows sustained elevations of ATP/Pi and PCr/Pi vs. sham across both post-stimulation periods (ATP/Pi overall F_1,89_ = 7.917, *p* = 0.006; PCr/Pi overall F_1,89_ = 28.382, *p* < 0.001). The first stimulation produces a biphasic ATP/Pi response—a drop at ~10 min post-end (F_1,89_ = 7.240, *p* = 0.009) followed by a peak ~40 min later (F_4,356_ = 3.700, *p* = 0.006). In contrast, PCr/Pi rises monotonically without an early dip. By the experiment’s end, phosphate ratios converge toward sham, consistent with a finite on-target window; cortisol declines more strongly after the second stimulation (F_1,14_ = 8.114, *p* = 0.013), reinforcing that same-day “double dosing” can both re-engage neurometabolic effects and extend endocrine modulation [167]. Practical implication: if the therapeutic goal is to pair stimulation with a behavior (e.g., exercise) or a physiologic challenge (e.g., mixed meal), a second session ~2 h after the first can stack two ~60 min GIR windows within a ~4–5 h treatment block.

With repeated daily dosing, the duration of effect increases even as some acute neurometabolic signatures adapt. In an 8-day, single-blind, crossover study of daily 1 mA, 20 min M1 stimulation, blood glucose (every 5 min for 70 min post-stimulation) is lower than sham on day 1 for ~50 min (mean difference ≈ 0.119 mmol/L, *p* = 0.031), and by day 8 the reduction persists across the entire 70 min window (treatment effect *p* = 0.009; timepoint p’s at 55/60/70 min = 0.007/0.019/0.045) without any change in insulin; cortisol/ACTH remain unchanged across days [166]. On day 1, ATP and PCr rise significantly vs. sham (both *p* < 0.001), but by day 8 these increases disappear, and ATP/PCr after tDCS are lower than on day 1 (ATP *p* = 0.001; PCr *p* < 0.001). Despite this attenuation, on day 8, there is a significant inverse correlation between PCr and glucose in the active condition (r = −0.642, *p* = 0.013). The dosing implication is twofold: (i) repetition lengthens the clinically useful glycemic window, even if absolute ATP/PCr boosts habituate; and (ii) within-course neurometabolic “set-point” may shift, so relative phosphate levels—and not their absolute increase from baseline—track glucose benefit late in the course. This argues for maintenance schedules (e.g., several weeks of near-daily sessions, then tapered boosters) and serial ^31^P-MRS as a response biomarker, while cautioning that loss of an early ATP/PCr surge does not equal loss of efficacy.

Montage and current also shape the latency and domain of benefit. In people with overweight/obesity, following a 4-week hypocaloric diet, right-DLPFC, 2 mA, 20 min, 20 sessions improved fasting plasma glucose (−7.8 mg/dL, *p* = 0.013) and Matsuda insulin sensitivity (+4.6 pmol^−1^·mmol^−1^, *p* = 0.002) without changing post-prandial glucose/insulin AUCs or β-cell indices [168,169]. This profile-tonic fasting improvement without acute meal test change suggests that higher current and prefrontal targeting deliver slower-onset, cumulative effects (likely via insulin sensitivity and/or basal hepatic output) that emerge over weeks, rather than the hour-scale windows seen with single-session M1. Conversely, network-targeted hypothalamus-anchored net-tDCS (25 min/session × 3 consecutive days) produces immediate behavioral effects—shorter stop-signal reaction time and lower sweet-calorie intake—yet no change in OGTT-derived insulin sensitivity ~20 h after the last session was noted [170]. The temporal inference is that behavioral and circuit-level modulation can precede metabolic change by >24 h and likely requires more sessions or longer follow-up to translate into measurable glycemic endpoints.

Taken together, the dosing–time course map is consistent and actionable: (a) single 20 min M1, 1 mA sessions create a predictable 1 h metabolic window beginning after an initial ~30 min lull, with a late peak around 3 h; (b) a second session ~115 min later reliably reopens that window; (c) near-daily repetition over ~1–2 weeks lengthens post-stimulation glycemic effects even as early ATP/PCr surges habituate; (d) higher-dose prefrontal protocols over 4 weeks yield cumulative fasting/insulin-sensitivity gains rather than acute post-meal changes; and (e) hypothalamus-network protocols show rapid behavioral but delayed metabolic trajectories, arguing for >3 sessions and longer metabolic follow-up. For diabetes trials, these kinetics suggest aligning session timing with physiologic needs (e.g., pre-exercise or high-insulin-resistance periods), testing double-session days to broaden coverage, embedding multi-week courses for prefrontal/network targets, and tracking both acute (hours) and cumulative (weeks) outcomes with parallel neurometabolic biomarkers to guide dose and schedule [165,166,167,168,169,170].

### 5.3. Near-Term Clinical Niches to Test

The neurometabolic and neurochemical signatures across studies [165,166,167,168,169,170] point to three immediately testable use cases that align with diabetes pathophysiology and can be implemented with concrete, evidence-anchored parameters. First, insulin-resistant prediabetes and early type 2 diabetes not using prandial insulin are prime candidates for an insulin-independent disposal augmentation strategy based on the motor cortex (M1) paradigm. Here, single 20 min, 1 mA anodal M1 sessions create a reproducible ~60 min window of increased glucose infusion rate (GIR) after an initial ~30 min lull, with a late peak ~200 min after onset, while circulating insulin and clamp glycemia remain unchanged. ATP/Pi and PCr/Pi show a dip→rebound on ^31^P-MRS that tracks disposal [165]. A same-day second session ~115 min after the first re-opens a second ~60 min GIR window and re-elevates ATP/Pi and PCr/Pi, with a more substantial late cortisol reduction [167]. Near-term trials in this niche should randomize adults with HbA1c ~6.0–7.9% and HOMA-IR/Matsuda-defined insulin resistance to (i) single-session days or (ii) “double-dose” days (two 20 min, 1 mA M1 blocks separated by 90–120 min) delivered 4–5 days/week for 4–8 weeks, time-aligned either before moderate exercise or during higher insulin-resistance windows (e.g., evening). Primary outcomes should capture insulin-independent effects: clamp M with tracers to separate hepatic glucose production vs. peripheral uptake, CGM time-in-range and mean glucose over the 1–6 h post-stimulation window, and fasting glucose/insulin; mechanistic secondaries include ^31^P-MRS (ATP/Pi, PCr/Pi) at baseline/early/late dosing, cortisol/ACTH, and ambulatory BP to mirror the acute endocrine and hemodynamic shifts seen in [165,167]. Safety monitoring should emphasize hypoglycemia (if on sensitizers), orthostatic symptoms, and autonomic signs, given the observed BP reductions in [165].

Second, obesity-linked insulin resistance with hedonic overeating (with or without prediabetes) maps to a behavior-plus-metabolism approach using proper DLPFC stimulation integrated with structured calorie restriction. In a 4-week program (5 weekdays/week; 2 mA, 20 min, 20 sessions), fasting glucose fell and Matsuda insulin sensitivity rose beyond diet alone, whereas post-prandial glucose/insulin AUCs and β-cell indices were unchanged [168,169]. These data argue for cumulative, tonic improvements (basal hepatic output/peripheral sensitivity) rather than acute meal handling. Near-term trials should enroll adults with BMI ≥ 30 kg/m^2^ (or ≥27 kg/m^2^ with dysglycemia) into a prespecified hypocaloric diet and randomize to active vs. sham right-DLPFC (2 mA, 20 min) for 4–6 weeks, optionally extending to maintenance boosters (weekly × 8–12 weeks). Endpoints should mirror observed gains and anticipated behavior-to-metabolism translation: fasting glucose/insulin, Matsuda index, CGM metrics, weight/body fat, and ad libitum “sweet-calorie” intake using standardized buffet paradigms; mechanistic readouts can include executive control (stop-signal reaction time) to link prefrontal engagement to adherence and dietary choice. Because meal AUCs did not separate in [168,169], studies should be powered primarily for fasting indices and CGM-based day-long exposure rather than isolated post-prandial peaks.

Third, stress-exacerbated hyperglycemia (with or without hypertension)—a typical phenotype in T2D—matches a neuroendocrine/autonomic modulation niche suggested by cortisol and ACTH reductions and BP lowering after M1 stimulation ([165], replicated for cortisol after the second session in [167]). Here, adults with documented glycemic deterioration during psychosocial stress or high perceived-stress scores can be randomized to M1 (1 mA, 20 min) courses (single or double-dose days as above) for 4–6 weeks, with CGM to quantify stress-linked excursions, salivary or plasma cortisol profiles to capture diurnal slope and area under the curve, and ambulatory BP/HRV as autonomic correlates. The mechanistic prediction—grounded in and [165,167]—is that dampening HPA tone and shifting autonomic balance will co-move with improved glycemia without requiring higher insulin. Given that an 8-day course showed glucose lowering without consistent hormone change [166], protocols should a priori test whether dose (single vs. double), montage, or session timing determines endocrine responsiveness and whether glycemic gains can persist as ^31^P-MRS surrogates “adapt.”

Finally, emerging hypothalamus-anchored, network-targeted stimulation offers a fourth, prevention-oriented niche centered on food choice and inhibitory control. After three 25 min sessions, Stop-Signal performance improved and sweet-calorie intake fell without change in OGTT-derived insulin sensitivity at ~20 h [170]. Near-term studies should therefore increase session number (≥10–15) and extend metabolic follow-up (≥4–12 weeks), embedding behavioral tasks during stimulation and coupling with dietary counseling. Outcomes should prioritize persistent changes in sweet-calorie intake, weight, CGM exposure, and liver fat, with OGTT/Matsuda as secondary metabolic readouts, acknowledging that behavioral gains likely precede measurable metabolic shifts at this target [170]. Together, these niches operationalize what the six studies show: M1 tDCS offers time-locked, insulin-independent metabolic windows suitable for disposal augmentation in dysglycemia [165,166,167]; right-DLPFC delivers multi-week fasting and sensitivity benefits alongside lifestyle change [168,169]; and hypothalamus-network stimulation provides a behavioral lever on hedonic intake that may require more sessions and longer horizons to manifest metabolic benefit [170].

### 5.4. Trial Blueprints (Mechanism-Anchored and Diabetes-Relevant)

Building directly on the kinetic and mechanistic signals in [165,166,167,168,169,170], three complementary randomized, double-blind, sham-controlled phase II programs can be launched now, each anchored to a specific brain–metabolism pathway and using endpoints that read out that pathway with minimal ambiguity.

#### 5.4.1. Program A-Insulin-Independent Disposal Augmentation (M1-Anchored)

Target population: adults with insulin-resistant prediabetes or early type 2 diabetes (HbA1c ~6.0–7.9%), stable on non-insulinotropic agents (or medication-naïve), without autonomic failure. Mechanistic premise: anodal M1 tDCS produces a time-locked rise in whole-body glucose uptake under clamp without raising insulin, alongside a characteristic ^31^P-MRS ATP/Pi and PCr/Pi dip→rebound that correlates with disposal and acute HPA/BP dampening [165], reproduced and extendable with a same-day “double dose” [167]. Arms and dose: (i) single-session days (1 mA, 20 min, anode over M1 as in [165], cathode supraorbital), (ii) double-session days (identical second block 90–120 min after the first, per [167]), and (iii) sham; 4–5 treatment days/week for 6–8 weeks. Session timing is prespecified to exploit the post-stimulation window observed in [165]: blocks start ~60–90 min before moderate exercise or during a patient’s higher insulin resistance period (e.g., evening) so the ~60 min GIR window and the later ~200 min peak bracket real-world glucose disposal. Primary endpoints: clamp M with tracers at baseline and week 6–8 to separate hepatic glucose production (EGP) from peripheral rate of disappearance (R_d), plus continuous glucose monitoring (CGM) time-in-range and mean glucose over 0–6 h after the first three sessions and at mid/late course, to capture the hour-scale effects that defined [165,167]. Secondary endpoints: fasting glucose/insulin, HOMA2-IR, ambulatory BP, salivary or plasma cortisol/ACTH profiles bracketing two treatment days, and standardized moderate-intensity exercise energy expenditure in the 2–4 h post-stimulation window. Mechanistic biomarkers and timing: ^31^P-MRS of ATP/Pi and PCr/Pi at baseline, +40 min, and +120–180 min after the first session (to capture the dip→rebound of [165]) and again early and late in the course (to observe adaptation as in [166]); correlation-planned analyses between phosphate ratios and disposal. Safety/operations: hypoglycemia surveillance if participants use sensitizers; orthostatic vitals around sessions given the BP drops in [165]; expectation/blinding checks; adherence captured by device logs.

#### 5.4.2. Program B-Tonic Insulin-Sensitivity and Fasting-Glycemia Improvement (Right DLPFC + Diet)

Target population: adults with obesity (BMI ≥ 30 kg/m^2^ or ≥27 with dysglycemia) with or without prediabetes, enrolled in a standardized hypocaloric diet. Mechanistic premise: 20 weekday sessions of right DLPFC (2 mA, 20 min; anode right DLPFC, cathode left DLPFC), adding fasting-glucose reduction and Matsuda insulin-sensitivity gains beyond diet, without separating post-prandial AUCs or β-cell indices [168,169]. Arms and dose: (i) active DLPFC per [168,169], (ii) sham, both on top of an identical diet; core phase 4–6 weeks, then a prespecified 8–12-week weekly booster phase to test durability. Primary endpoints: change in fasting plasma glucose and Matsuda index from a standardized mixed-meal tolerance test at weeks 4–6; CGM-derived mean glucose and time-in-range across each treatment week to privilege cumulative exposure rather than isolated peaks (reflecting [168,169]). Secondary endpoints: body weight and fat mass, fasting insulin and HOMA2-IR, mixed-meal glucose/insulin AUCs (hypothesized neutral as in [168,169]), and ad libitum sweet-calorie intake from a standardized buffet paradigm. Mechanistic/behavioral anchors: stop-signal reaction time tasks during a subset of sessions to index executive control that plausibly mediates dietary adherence; optional ^31^P-MRS in a mechanistic subgroup to test whether DLPFC courses also show phosphate-ratio adaptation akin to [166]. Safety/operations: dietitian-supervised intake; stable concomitant medications; blinding integrity checks because expectancy can influence eating behavior.

#### 5.4.3. Program C-Hedonic Intake Control via Hypothalamus-Network Targeting (Net-tDCS)

Target population: adults with overweight/obesity (with or without prediabetes) who report high sweet-calorie intake. Mechanistic premise: three sessions of network-targeted hypothalamus-anchored tDCS improved inhibitory control. They reduced sweet calories without shifting OGTT-derived insulin sensitivity 20 h later [170], implying behavioral change precedes measurable metabolic change. Arms and dose: (i) anodal net-tDCS to the hypothalamus appetite-control network (multielectrode montage as in [170]), (ii) sham; session count extended to ≥12–15 sessions (25 min each over 3–5 weeks) to allow behavior→metabolism translation, with behavioral tasks (e.g., Stop-Signal) performed during stimulation as in [170] to maximize network engagement. Primary endpoints: change in sweet-calorie intake during standardized ad libitum meals at weeks 2 and 5 and change in CGM metrics (mean glucose, time-in-range, glycemic variability) over the whole course; weight and liver fat (MRI-PDFF) at baseline and week 12 to index downstream metabolic benefit. Secondary endpoints: OGTT glucose/insulin and Matsuda at weeks 5 and 12 (registered as secondary because [170] showed no shift at ~20 h); craving scales collected pre/post-selected sessions to test dissociation between subjective desire and observed intake reported in [170]. Mechanistic adjuncts: resting-state fMRI at baseline and post-course in a subgroup to replicate the hypothalamic connectivity–behavior coupling of [170]; optional ^31^P-MRS to explore whether network-level protocols show neurometabolic signatures comparable to M1 courses.

Cross-program methods are held in common. All programs use centralized randomization with sex and baseline insulin resistance strata, intention-to-treat mixed-effects analyses with prespecified contrasts at mechanistically informative timepoints (e.g., 0–6 h post-M1 sessions), and harmonized safety monitoring. Session ramp-up/down and electrode placement replicate the source protocols (M1: 1 mA, 20 min [165,166,167]; right DLPFC: 2 mA, 20 min, 20 sessions [168,169]; hypothalamus network: multielectrode, 25 min [170]). Concomitant medications remain stable; insulin and sulfonylureas are excluded or dose-locked in Program A to avoid confounding insulin-dependent effects, aligning with the insulin-independent mechanisms in [165,166,167]. Each protocol prespecifies mediation analyses linking neurometabolic signals (^31^P-MRS ATP/Pi, PCr/Pi) or circuit engagement (hypothalamic connectivity, inhibitory-control performance) to glycemic endpoints, reflecting the correlations and adaptations documented in [165,166,167] and the behavior–network coupling in [170]. Finally, because [166] showed persistence of glycemic benefits despite attenuation of ATP/PCr boosts by day 8, all programs include late-course biomarker sessions to distinguish loss of a neurometabolic surge from loss of clinical effect and booster phases to map durability and decay after the initial course.

## 6. Conclusions

Across heterogeneous cohorts with diabetes—predominantly type 2 with painful peripheral neuropathy—tDCS shows target-specific, clinically meaningful benefits for complications and functioning. Anodal M1 delivers the most consistent analgesia and mobility gains; prefrontal (DLPFC) protocols, especially when coupled with cognitive training, improve working memory and anxiety; combined M1 + DLPFC courses can broaden effects to sleep quality and health-related quality of life; and occipital stimulation may acutely enhance visual performance in proliferative retinopathy. Protocols were generally safe and well-tolerated. Reported pain, sleep, and affect benefits were sustained for up to three months in the longest studies, suggesting moderate durability of response that warrants longer-term follow-up. In contrast, objective metabolic outcomes (fasting glucose, HbA1c, insulin sensitivity) are infrequently reported in diabetic samples. Early neuro-metabolic findings—such as insulin-independent glucose disposal and cerebral high-energy phosphate shifts—arise mainly from non-diabetic or mixed cohorts and should be considered hypothesis-generating only. Accordingly, tDCS should presently be viewed as a promising adjunct for diabetes-related complications rather than a validated tool for glycemic control. Priorities for future trials include adequately powered, rigorously sham-controlled studies that (i) match cortical targets to patient phenotypes, (ii) optimize dose/schedule (including potential “double-dose” paradigms), and (iii) embed metabolic endpoints and mechanistic biomarkers (e.g., clamp-based insulin sensitivity, CGM metrics, ^31^P-MRS) alongside patient-centered outcomes.

## Figures and Tables

**Table 1:** Study inclusion and exclusion criteria.

Domain	Inclusion Criteria	Exclusion Criteria
Population	Humans with diabetes (any type, age, sex). Mixed cohorts eligible only if data for the diabetic subgroup were extractable.	Non-diabetic populations; mixed cohorts without separable diabetic data.
Intervention	tDCS, as stand-alone or adjunct. Any montage, dose, schedule.	Studies where tDCS was not used; mixed-modality neuromodulation where tDCS effects could not be isolated.
Comparator/Design	Randomized or non-randomized interventional designs, parallel or crossover; single-arm pre–post; single-case interventional reports.	Non-interventional designs (narrative/systematic reviews, meta-analyses, editorials, letters, opinions).
Outcomes	Any reported clinical/functional endpoints (e.g., pain, cognition/affect, sleep/QoL, vision). Not used as screening filters.	-
Timeframe	Publications dated 1 January 2008–31 August 2025; final database access Aug 2025.	Outside date range.
Language	Any language.	-
Publication type	Full-text articles.	Conference abstracts without an accompanying full text.

**Table 2:** Studies included in review.

Study	Population/Condition	Design	N (Total/per Group)	Montage and Dose	Session Plan	Comparator(s)	Primary Outcomes	Key Findings	Follow-Up	Adverse Events
Alipour, A. and Mohammadi, R., 2024 [60], Iran	Type 2 DM with neuropathic pain	Double-blind RCT; 4 arms (M1, F3, M1 + F3, sham)	48 total; 12 per arm	Anodal M1 (L/R) and/or F3; 2 mA	40 min/session; 12 sessions; 3×/wk	Sham	Pain (SF-MPQ-2) at pre, post, 2-mo FU	All active ↓ pain vs. sham post (*p* < 0.01) & at 2 mo (*p* < 0.001). Post: M1 < F3 (better; Δ = −7.58, *p* = 0.025). No diff M1 vs. M1 + F3; active arms equal at 2 mo.	2 months; benefits maintained	Did not report any significant adverse or harmful side effects
Aksu et al., 2025 [61], Turkey	Type 2 DM with painful DPN	Randomized, triple-blind, sham-controlled; parallel	28 total; 1:1 active:sham	Anode F3, cathode F4; 2 mA	20 min/session; 5 consecutive weekdays; with adaptive N-back WMT	Sham	WM (verbal d′ 2-back; Corsi forward); pain (VAS, S-LANSS, NEPIQOL); psych (BDI, BAI)	↑ Verbal and visuospatial WM in active at 1 mo (*p* ≤ 0.011, Bonferroni). No pain benefit. Anxiety ↓ in active (BAI, *p* = 0.001). No effect on depression.	1 month; WM and anxiety benefits persisted	Did not report any significant adverse or harmful side effects
de Venecia, A.B.F. 3rd and Fresnoza, S.M, 2021 [62], Philippines	Proliferative diabetic retinopathy (PDR)	Randomized, sham-controlled	22 total; 11 tDCS, 11 sham	Cathode Oz (V1), anode right shoulder; 1 mA	10 min; single session	Sham	Visual acuity (LogMAR), Number acuity (reaction time (RT), accuracy)	↓ LogMAR both eyes in tDCS (*p* ≤ 0.020); RT ↓ markedly (*p* ≤ 0.001); accuracy ~ceiling; sham no change.	Immediate post only	- One patient in the sham group reported mild headache, neck fatigue, and increased heart rate after stimulation - These symptoms resolved on their own, and the participant was able to go home once they subsided - Patients in the cathodal tDCS (treatment) group did not report any side effects
Ferreira et al., 2020 [63], Brazil	Diabetic polyneuropathy	Randomized, sham-controlled pilot	20 total; 10 active, 10 sham	Anode C3 (M1), cathode Fp2; 2 mA	20 min; 5 consecutive days	Sham	SF-36 QoL (eight domains; composites)	Active > sham for total SF-36 and physical domains (physical functioning, bodily pain). TUG and 6MWT improved only in active.	1 and 2 weeks post; benefits present	Did not report any significant adverse or harmful side effects
Wu et al., 2016 [64], Taiwan	DPN (Dyck 2a/2b); matched controls	Within-subject crossover (active vs. sham), order counterbalanced	16 patients (plus 16 controls for baseline)	Anode right DLPFC; cathode left cheek; 2 mA	15 min; 2 sessions ≥ 24 h apart (active vs. sham)	Sham	Visuospatial WM (computerized Corsi) under interference/no-interference conditions	Largest gain after tDCS in interference condition (span 3.59→4.22). No such gain on sham day. Baseline VSWM correlated with NCV; post-tDCS low performers caught up.	Immediate post within-session	Did not report any significant adverse or harmful side effects
Mohomad et al., 2015 [65], USA	Case: plantar fasciitis heel pain in T2DM	Single-patient case report	n = 1	Anode M1 leg area (C2), cathode supraorbital; 2 mA	20 min; 5 consecutive days	None	Pain (VAS), Pain anxiety (PASS-20)	VAS 7.9→2.3 post→1.7 at 1 wk; PASS-20 40→31; stopped opioids after day 2.	1 week post; maintained	- Mild tingling under the electrodes (during stimulation) - Mild, transient fatigue (after the fourth day of stimulation) - No significant adverse effects were noted overall
Kim et al., 2013 [66], South Korea	Painful diabetic polyneuropathy (PDPN)	Randomized, sham-controlled; three arms (M1, DLPFC, sham)	60 completers (of 72 randomized)	Anode C3 (M1) or F3 (DLPFC); cathode supraorbital; 2 mA	20 min; 5 consecutive days	Sham	Pain (VAS); secondary: CGI, Pressure pain threshold, anxiety, sleep, BDI	M1: ~34% VAS ↓ (5.75→3.80), sus-tained 2 and 4 wks; 65% achieved ≥30% pain relief. DLPFC: ~22% ↓, not sustained; Sham: ~14% ↓. CGI and PPT improved most in M1.	2 and 4 weeks; sustained for M1	- Total incidence: six adverse events across all three groups (M1, DLPFC, and sham) Types of side effects: - Headache—three patients (two in the M1 group, one in the DLPFC group) - Itching under the electrodes—three patients (one in each group) Dropouts due to adverse effects: - One participant withdrew because of a mild headache Overall tolerance: - All other participants tolerated tDCS well, and no significant or serious adverse effects were observed - The overall rate of mild adverse events was 8.33%, notably lower than in other tDCS studies on chronic pain
Rahmy et al., 2018 [67], Egypt	Diabetic peripheral neuropathy pain	Randomized parallel group (tDCS vs. TENS)	40 total; 20 per arm	tDCS: anode M1, cathode supraorbital; up to 1 mA	20 min; 3×/wk for 2 months	TENS (peripheral)	Neuropathy Pain Scale (NPS)	Both groups improved (~53.5%); no between-group difference.	Post-treatment only	Mild, short-lived discomfort: - At the start of stimulation, most patients felt a *slight itching sensation* under the electrodes, which disappeared within a minute or less Occasional transient sensations: - Some participants might feel *dizziness or vertigo* if the current was suddenly increased or decreased
ElSayed et al., 2020 [68], Saudi Arabia	Diabetic peripheral neuropathy (mild–moderate pain)	Pre–post single-arm (no control)	20	Anode M1, cathode supraorbital; up to 1 mA	20 min; 3×/wk for 2 months	None	Neuro-QoL; Neuropathy Pain Scale (NPS)	Large ↓ across all NPS pain qualities (≈50–75%) and ↑ QoL domains (e.g., applied cognition +119%).	Post-treatment only	Did not report any significant adverse or harmful side effects
Alipour, A. and Mohammadi, R, 2023 [69], Iran	Type 2 DM with neuropathic pain	Double-blind RCT; 4 arms (M1, F3, M1 + F3, sham)	48 total; 12 per arm	Anodal M1 (L/R) and/or F3; 2 mA	40 min/session; 12 sessions; every other day	Sham	Sleep (PSQI), quality of life (SF-36 composite) at post, 1 mo, 3 mo	Combined M1 + F3 superior to sham for PSQI; M1-only and F3-only not > sham. For SF-36, combined > sham and > F3-only; M1-only not > sham. Improvements maintained through 3 mo.	1 and 3 months; maintained	Did not report any significant adverse or harmful side effects
Alipour, A. and Mohammadi, R, 2023 [70], Iran	Type 2 DM with neuropathic pain	Randomized; 4 arms (M1, F3, M1 + F3, sham)	48 total; 12 per arm	Anodal M1 (L/R) and/or F3; 2 mA	40 min/session; 12 sessions; 3×/wk	Sham	Psychological distress (DASS-42) at post, 1 mo, 3 mo	All active arms ↓ distress vs. sham (*p* < 0.01); no differences among active arms; benefits stable through 3 mo.	1 & 3 months; maintained	Did not report any significant adverse or harmful side effects

**Table 3:** Risk of bias assessment.

Study	Bias Arising from the Randomization Process	Bias Due to Deviations from Intended Interventions	Bias Due to Missing Outcome Data	Bias in Measurement of the Outcome	Bias in Selection of the Reported Result
Alipour, A. and Mohammadi, R., 2024 [60], Iran	Low risk	Low risk	Low risk	Some concerns	Some concerns
Aksu et al., 2025 [61], Turkey	Low risk	Low risk	Low risk	Low risk	Some concerns
de Venecia, A.B.F. 3rd and Fresnoza, S.M, 2021 [62], Philippines	Low risk	Low risk	Low risk	Low risk	Some concerns
Ferreira et al., 2020 [63], Brazil	Low risk	Low risk	Low risk	Some concerns	Some concerns
Wu et al., 2016 [64], Taiwan	Some concerns	Some concerns	Low risk	Low risk	Some concerns
Mohomad et al., 2015 [65], USA	High risk	Some concerns	High risk	High risk	Some concerns
Kim et al., 2013 [66], South Korea	Some concerns	Low risk	Low risk	Low risk	Some concerns
Rahmy et al., 2018 [67], Egypt	Some concerns	High risk	Low risk	High risk	Some concerns
ElSayed et al., 2020 [68], Saudi Arabia	High risk	High risk	Low risk	High risk	Some concerns
Alipour, A. and Mohammadi, R, 2023 [69], Iran	Some concerns	Low risk	Some concerns	Low risk	Some concerns
Alipour, A. and Mohammadi, R, 2023 [70], Iran	Low risk	Low risk	Some concerns	Some concerns	Low risk

**Table 4:** Metabolic and neurochemic tDCS studies.

Study	Sample/Population	Design/Stimulation Parameters	Target Brain Area	Outcome Measures	Main Results
Binkofski et al., 2011 [165], Switzerland	n = 15 healthy young men (mean age 24 y; BMI 23.2)	Randomized, sham-controlled, crossover; anodal tDCS 20 min, 1 mA	Left primary motor cortex (M1)	Hyperinsulinemic–euglycemic clamp, ^31^P-MRS (ATP, PCr/Pi), cortisol, ACTH, blood pressure	↑ Glucose uptake vs. sham (*p* = 0.001); no change in serum insulin or plasma glucose; transient ↓ then ↑ in ATP/Pi and PCr/Pi; ↓ cortisol and ACTH (*p* = 0.004); ↓ BP (*p* < 0.05)
Kistenmacher et al., 2017 [166], Germany	n = 14 healthy men (mean age 25 y; BMI 22.6)	Sham-controlled, single-blind, crossover; 8 daily sessions, 20 min, 1 mA	Motor cortex (M1)	Blood glucose, insulin, cortisol, ACTH, ^31^P-MRS	↓ Blood glucose (−0.119 mmol/L; *p* = 0.031) after stimulation, lasting 50–70 min; no change in insulin; day 8 sustained ↓ glucose (*p* = 0.009); early ↑ ATP and PCr (*p* < 0.001), later normalization; negative correlation PCr–glucose (r = −0.642)
Wardzinski et al., 2019 [167], Germany	n = 15 healthy men (mean age 25 y; BMI normal)	Randomized, sham-controlled, cross-over; two 20 min 1 mA sessions, 115 min apart	Motor cortex (M1)	Glucose infusion rate, ^31^P-MRS (ATP/Pi, PCr/Pi), cortisol, ACTH	↑ Glucose infusion rate after both sessions (*p* = 0.042, 0.013); ↑ ATP/Pi and PCr/Pi (*p* < 0.01); ↓ cortisol after 2nd stimulation (*p* = 0.013); no change in ACTH or insulin
De Araujo et al., 2022 [168], Brazil	n = 28 (mean age 37.6 y; BMI 25–35; 79% obese)	Randomized, double-blind; 20 sessions, 2 mA, 20 min; 4 weeks + hypocaloric diet	Right DLPFC (anode)–Left DLPFC (cathode)	Fasting glucose, insulin, HOMA-IR, Matsuda Index (MISI), HbA1c, glycated albumin	↓ Fasting glucose (−7.8 mg/dL, *p* = 0.013) and insulin (−7.7 µIU/mL, *p* = 0.013); ↑ MISI (+4.6, *p* = 0.002); no change in HbA1c, postprandial AUC, or β-cell indices
De Araujo et al., 2019 [169], Brazil	n = 28 overweight/obese adults (79% obese; some with T2D or IGT)	Randomized, double-blind; 20 sessions, 2 mA, 20 min, 4 weeks	Right DLPFC	Fasting glucose, insulin, MISI, ISI, Disposition Index	↓ Fasting glucose (−7.8 mg/dL vs. −0.9 sham); ↑ MISI (*p* < 0.05); no change in ISI, DI, or AUCs; improved insulin sensitivity independent of weight loss
Ester-Nacke et al., 2025 [170], Germany	n = 44 (mean age 36 y; BMI 30.6; overweight/obese adults)	Randomized, double-blind, parallel; three sessions, 25 min, 12-electrode net montage	Hypothalamus appetite-control network	Oral glucose tolerance test (oGTT), fasting glucose, insulin, HbA1c, ISI Matsuda	No significant changes in glucose, insulin, ISI, or HbA1c; anodal tDCS ↓ sweet food intake (*p* = 0.037) and improved inhibitory control (↓ SSRT); ↑ hypothalamic connectivity on fMRI

Abbreviations: ACTH, adrenocorticotropic hormone; AUC, area under the curve; ATP, adenosine triphosphate; BMI, body mass index; BNDF, brain-derived neurotrophic factor; BP, blood pressure; C-peptide, connecting peptide; DI, disposition index (β-cell function index); DLPFC, dorsolateral prefrontal cortex; fMRI, functional magnetic resonance imaging; HbA1c, glycated hemoglobin; HD-tDCS, high-definition transcranial direct current stimulation; HOMA-IR, homeostatic model assessment for insulin resistance; IGT, impaired glucose tolerance; ISI, insulin secretion index; ISI (Matsuda), insulin sensitivity index (Matsuda method); LMTT, liquid meal tolerance test; M1, primary motor cortex; MISI, Matsuda insulin sensitivity index; ^31^P-MRS, phosphorus magnetic resonance spectroscopy; net-tDCS, network-targeted transcranial direct current stimulation; PCr, phosphocreatine; Pi, inorganic phosphate; PG, plasma glucose; RVM, rostroventromedial medulla; SSRT, stop-signal reaction time; T2D, type 2 diabetes; tDCS, transcranial direct current stimulation; WM, working memory.

## Data Availability

No new data were created or analyzed in this study. Data sharing does not apply to this article.
